# From an Invasive Weed to an Insecticidal Agent: Exploring the Potential of *Lantana camara* in Insect Management Strategies—A Review

**DOI:** 10.3390/ijms252312788

**Published:** 2024-11-28

**Authors:** Randeep Kumar, Niraj Guleria, Mudagadde G. Deeksha, Nisha Kumari, Ravendra Kumar, Arun Kumar Jha, Neha Parmar, Pritam Ganguly, Eloisa Helena de Aguiar Andrade, Oberdan Oliveira Ferreira, Mozaniel Santana de Oliveira, Chandini

**Affiliations:** 1 Department of Soil Science and Agricultural Chemistry, Bihar Agricultural University, Sabour, Bhagalpur 813210, Bihar, India; randeep.agchem@gmail.com (R.K.); nkpsanvisri@gmail.com (N.K.); jhaak_ss@rediffmail.com (A.K.J.); pritam0410@gmail.com (P.G.); 2 Mountain Agricultural Research and Extension Station, CSKHPKV, Salooni 176320, Himachal Pradesh, India; nirajguleria333@gmail.com; 3 ICAR-Directorate of Weed Research, Jabalpur 482004, Madhya Pradesh, India; deekshamudagadde@gmail.com; 4 Department of Chemistry, College of Basic Science and Humanities, G.B. Pant University of Agriculture and Technology, Pantnagar 263145, Uttarakhand, India; ravichemistry.kumar@gmail.com; 5 Department of Veterinary Public Health and Epidemiology, Guru Angad Dev Veterinary and Animal Sciences University, Ludhiana 141004, Punjab, India; npvet36@gmail.com; 6 Laboratório Adolpho Ducke—LAD—Museu Paraense Emílio Goeld, Av. Perimetral, 1901-Terra Firme, Belém 66077-830, PA, Brazil; eloisa@museu-goeldi.br (E.H.d.A.A.); oberdan@museu-goeldi.br (O.O.F.); 7 Department of Agronomy, Bihar Agricultural University, Sabour, Bhagalpur 813210, Bihar, India

**Keywords:** *Lantana camara*, insecticide, molecular docking, contact action, fumigant

## Abstract

*Lantana camara* is weed with a wide range of chemical constituents, including primary and secondary metabolites such as alkaloids, flavonoids, tannins, saponins, and terpenoids. These compounds contribute to its medicinal and pesticidal potential. The essential oils and different solvent fractions derived from *L. camara* exhibit notable variations in their respective chemical compositions across various plant parts, spatial distributions, and interspecific comparisons. The principal components, notably lantadene A, lantadene B, lantadene C, lantadene D, β-caryophyllene, α-humulene, and several others, constitute a significant portion of the essential oil derived from the leaves and flowers. Researchers have discovered that *L. camara* oil exhibits potent insecticidal activity against a range of pests, with variations in potency observed across different seasons due to changes in chemical composition. In addition to the essential oils, solvent extracts of *L. camara*, primarily methanolic extracts of the leaves of this species, demonstrate considerable potential as fumigant and contact toxins for stored grain pests. However, these have been comparatively less characterized with respect to their insecticidal properties, particularly in comparison to the essential oils. Molecular docking studies have demonstrated that phytochemical compounds present in the plants interfere with the activity of several enzymes that are responsible for the growth and survival of insects. For example, compounds such as β-caryophyllene and linalool exhibited a high binding affinity to AChE, thereby enhancing its neurotoxic effects. In conclusion, this review identifies *L. camara* as a natural insecticide with a complex set of modes of action attributed to its rich phytochemical profile. The integration of traditional knowledge with modern molecular techniques might expose avenues for the sustainable management of pests and control, ultimately making *L. camara* a key resource for such applications. Further studies are necessary to characterize such bioactive compounds and their uses in controlling pests in agricultural operations.

## 1. Introduction

*Lantana camara* derives from Latin word “lento”, meaning to bend [1]. In 1753, the binomial name of the *L. camara* species was first described and acknowledged by Linnaeus. It belongs to the verbenaceae family and Lamiales order with 600 species of perennial flowering plants [2] (Table 1). *L. camara*, known as a versatile species, is valued for its medicinal properties and ornamental appeal. According to Gaur [3], *L. camara* thrives in tropical, subtropical, and temperate regions at elevations up to 2000 m. *L. camara* is a versatile species that can grow in an extensive range of habitats and in diverse types of soils. It is mostly found in open, unshaded areas such as wastelands, rainforest edges, beachfronts, shrub/scrub lands, agricultural or urban areas, grasslands, riparian zones, and disturbed forests belonging to logging or fire activities. The *L. camara* species also flourishes in undisturbed areas like roadsides, railway tracks, and canals. Human activities often promote the spread of *L. camara* and its invasion into new areas. The species also has the ability to grow in diverse climatic conditions. It can tolerate both high rainfall regions exceeding 200 inches annually and drier areas with as little as 30 inches of rainfall. Lantana is easily propagated from cuttings or seeds, which are often dispersed by birds [3]. Its ability to regenerate quickly after cutting, trampling, or burning contributes to its widespread distribution and invasive nature in many regions (Table 2).

*L. camara* Linn, an indigenous species of Central America, South America, and the Caribbean Islands [5], has become introduced geographically in many regions worldwide. It is often found in Mexico, Brazil, Jamaica, Florida, and Trinidad. This species is commonly established in tropical and subtropical regions of India, America, Africa, and Australia. *L. camara* has also been introduced and found in some parts of Africa and India [6]. *L. camara* is known by different names across India. This reflects the plant’s widely distributed and cultural significance throughout the Indian subcontinent. In India, the genus Lantana is delineated by four species, namely, *L. indica*, *L. camara*, *L. veronicifolia*, and *L. trifolia* [7]. Among these, *L. camara* is mainly problematic and has been recorded worldwide to be one of the worst invasive weeds posing a significant risk to its micro-ecosystem at several scales. Throughout tropical and subtropical India, it is present in disturbed habitats, ranging from foothills to seacoast. The Lantana species is considered as complex, having more than one violent weedy type, as represented in India. In contrast, *L. trifolia*, *L. indica*, and *L. veronicifolia* are less violent and are present in confined patches. They are believed to have been introduced to the country but have become naturalized on their own [8]. *L. camara* is commonly known for having a strong, standing tree, including a triangular stem. It is a versatile shrub that can thrive in various climates. Although it can be a beautiful addition to gardens with its vibrant flowers, it is also observed as a highly invasive species in many regions. Lantana can grow up to 1–3 m in height. It has a spreading habit and can reach well up to 2.5 m. The leaves are opposite, acute or subacute with serrated edges. They are green and rough on the upper surface, while the undersides are often fuzzy. Lantana is popular for its fancy flowers that are typically clustered in small, rounded heads. The flowers can be of various colours, including orange, white, red, pink, and purple, and often change colour over time. Almost year-round, yellowish flowers are present on the axillary head. Small, corolla-shaped calyces and tubes, up to 6 to 7 mm long, are observed on irregular lobes. The flowers are arranged in clusters called inflorescences, which are found in pairs in the axils of many leaves. Together, the dome-shaped inflorescences mainly contain 20 to 40 tiny flowers. The plant has strong roots that enable it to spread rapidly and regenerate even after repeated cuts. Fruits are drupaceous and greenish-black in colour. Green coloured seeds are formed when unripe, and when ripe, they become purplish-black coloured seeds [7,9].

*L. camara* has been introduced as a noxious weed in many parts of India. It is ranked among the top ten noxious weeds worldwide. This is why it is known as a poisonous plant [10]. Because of its prolific plant type and dispersal nature, aggressive growth, dense thickets, and allelopathic properties, it has caused widespread ecological disruption. By virtue of its gregarious nature, the species alters the terrestrial ecosystem. *L. camara* forms solid groves that eradicate the natural plant species, leading to habitat degradation and loss of biodiversity. The displacement of native understory plants by Lantana leads to reduced overall plant biomass [10]. The allelopathic nature of the Lantana plant leads to the minimization of the growth of other species, further reducing plant diversity [11]. Allelopathic features of *L. camara* facilitate it to live through a secondary progression and act as monospecific groves. Lantana’s allelopathic properties hinder the natural succession process, preventing the establishment of native species, such as *Christella dentata*, *Lolium multiflorum* L., and *Morrenia odorata* L., and also many crops like wheat, soyabean, and corn [11]. *L. camara is* highly inflammable, leading to the altered fire regime of the forest. In many parts of India, *L. camara* is considered as a major problem in agricultural areas as it competes with flora, fauna, and pastures, reducing yields and disturbing livestock health [11]. In addition, it has also been reported that eating the green fruit has been identified to be lethal in India, while the ripe fruit of the Lantana species are commonly safe to eat. As a leading threat to biodiversity, *L. camara* can outcompete native species, hybridize with them, and introduce diseases. Its toxic compounds, known as lantadene A and B [10], have been reported to be harmful to grazing animals like goats, sheep, horses, and cattle, causing conditions such as hepatotoxicity, photosensitivity, and pink muzzle disease [12]. Therefore, *L. camara*’s invasive nature and toxic properties pose a serious threat to India’s natural ecosystems, resulting in the wide diversity loss of native species and consequently, the alteration of the ecosystem’s structure and function [13].

*L. camara* is one of the multifaceted medicinal plants having a wide range of traditional and modern applications that underscore its importance in ethnobotany and pharmacology as well as in pesticidal efficacy. Its leaves have been in use for many years within most cultures in their various applications of therapeutic uses. The traditional preparation usually involves drinking the leaves as some form of tea to treat ailments like tetanus, cough, and malaria, while leaf extracts in topical applications are used for wound healing. The whole plant, as used traditionally, is often utilized in the treatment of bronchitis and stomach-ache. The bioactive compounds present in *L. camara* had shown promise to be a cardiotonic, and thus would be used for cardiovascular health. In addition, the leaves have been used to treat rheumatism, ulcers, and cuts. This indeed underlines *L. camara* as an integral part of herbal medicine. The scientific studies described the pharmacological nature of the plant. Some reports have mentioned that the extracts exhibited significant antifungal activities. Other research indicates some antibacterial activities against *Escherichia coli*, *Bacillus subtilis*, and *Pseudomonas aeruginosa*. *L. camara* also showed potent insecticidal activity which was evidenced by published articles. Also, nematocidal activities have been reported in the polar fractions of leaf extracts. In addition, the wide variation of the plant’s insecticidal activity indicates that *L. camara* could be an environmentally friendly option for sustainable agriculture practices, especially as an alternative to synthetic pesticides, in integrated pest management [14,15,16,17,18].

The collective body of evidence does support *L. camara* as a useful medicinal plant with broad therapeutic usage and as a potent insecticide as well. Its bioactive compounds hold promise to be further researched into developing a management tool for many pests, and the effectiveness with which it controls pests in its environment places it as a candidate of high importance for sustainable agriculture. Therefore, this review highlights a comprehensive review on the phytochemistry of the plant as well as its potent insecticidal efficacy. We elucidated not only the bioefficacy but the mechanism of action responsible for its potential pesticidal efficacy. This review also highlights the vital pest’s enzymes that could be a potential target for *L. camara’s phytoconstituents* for significant insecticidal assay, elucidated via molecular docking studies.

## 2. Insights into the Pharmacological Action of *L. camara*

*L. camara*, considered as a medicinal plant, is distinguished for its various chemical constituents, including primary and secondary metabolites such as alkaloids, tannins, glycosides, saponins, carbohydrates, resins, steroids, cardiac glycosides, coumarins, phenols, flavonoids, terpenoids, and anthraquinones, which furnish its therapeutic potential. Many parts of *L. camara* have been studied for the assessment of its phytochemical constituents. Many scientists have reported that the leaf extracts have a preponderance of phytoconstituents including alkaloids, flavonoids, tannins, triterpenoids, saponins, and glycosides [8,12]. Even though the leaves of *L. camara* have been studied extensively in phytochemical studies, studies on stem and fruit extracts have also been performed, reporting the extract to be rich in saponins, flavonoids, tannins, and terpenoids. Furthermore, the presence of oleanolic acid has also been established in its roots [13]. *L. camara* is naturally enriched with monoterpene- and sesquiterpene-dominated essential oils and is commercially famous as Lantana oils. Worldwide, *L. camara* L. has been used as a therapeutic plant to cure many diseases [14]. The leaves of the plant have been used as tea, after boiling, for the treatment of tetanus, cough, and malaria, while the leaf extracts have been made into lotions that can also be used to heal wounds [15]. The whole plant has been used for treating bronchitis and stomach-ache in Ghana [14]. It has been reported that an important steroid extracted from the leaves, lancamarone, possesses the potential to treat cardiac conditions, as a cardiotonic substance. Conventionally, the leaves of lantana might be used as a supplement for abdominal pains. In many regions of Asia, the plant leaves have been reported to be used to treat ulcers, cuts, and rheumatisms. *L. camara*, considered as a medicinal plant, holds crucial antifungal potential [16]. Ganjewala et al. [2] screened its antifungal properties against *Alternaria* sp. In addition, they also reported that the leaves and flowers of the plants possess antibacterial potential against *Bacillus subtilis*, *P. aeruginosa*, and *E. coli*. An ethanolic extract of the leaves and roots of *L. camara* was reported to have antibacterial activity against *P. vulgaris*, *S. aureus*, *P. aeruginosa*, *E. coli*, *V. cholareae*, and two more resilient strains, *E. coli* and *S. aureus.* The ethanolic and ethyl acetate extracts of *L. camara* possess the potential for antipyretic activity. The estimation of the anthelmintic potential in the ethanolic extract from the stem of *L. camara* resulted in a positive response against *P. posthuman* [13,15,17]. Kumar [19] reported in his study that methanol and ethanol fractions extracted using the leaves and flowers of *L. camara* have potent mosquito larvicidal activity. Ahmad [20] examined *L. camara*’s nematocidal property and reported polar fractions of leaf extracts to possess the action against *Meloidogyne incognita* under in-vitro conditions. It was revealed that camaric acids and lantanilic acids, along with oleanolic acid, possessed significant nematocidal activities. *L. camara* shows solid insecticidal behaviour against a diverse range of insects. Furthermore, Rajashekar [17] suggested that the *T. castaneum* and *S. oryzae* species were found less susceptible compared with *C. chinensis*. A further study has also revealed the potential of *L. camara* to possess insecticidal, anti-ovipositional, and antifeedant activities [18] (Table 3).

## 3. Pesticidal Bioefficacy of *Lantana* spp.

**Figure 1:**
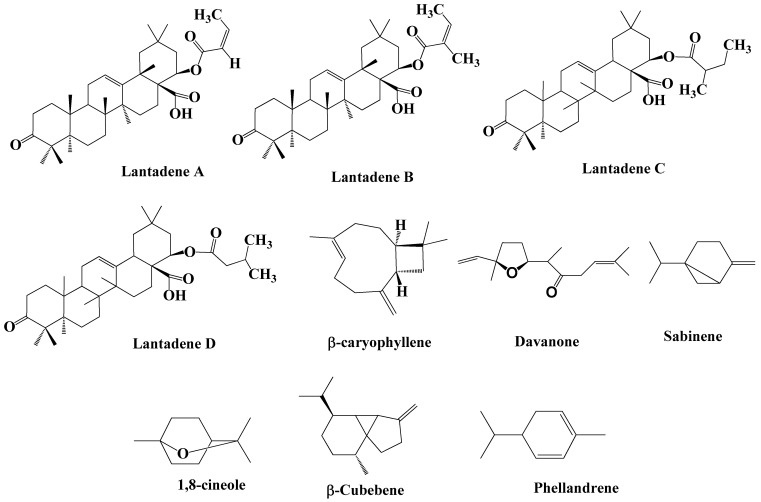
Figure representing dominant volatile and non-volatile phytochemicals reported in extracts and essential oils of *L. camara*.

The growing world population and the resultant demand for food dictate the use of synthetic chemicals as a swift and uncomplicated means of controlling pests in fields and stored grains. The over-reliance on synthetic insecticides to control these pests has led to significant detrimental effects on human health, the environment, and the development of resistance in these notorious pests [25,26]. This problem, coupled with the increased demand for organically produced food, has led to alternative approaches. Plant families with bioactive compounds have been used to manage different insect pests [27,28]. Phytocompounds such as essential oils, flavonoids, alkaloids, glycosides, esters, and fatty acids have caused anti-insect effects in different ways, viz., as repellents, feeding deterrents/antifeedants, toxicants, growth retardants, chemo-sterilants, and attractants [28]. Plants belonging to Verbenaceae, such as *Aloysia*, *Lippia*, and *Stachytarpheta*, including *Lantana,* have been known for their pharmacological properties [29], but there is a lack of studies investigating their insecticidal properties. Essential oils and other phytochemicals of *Lantana* spp., extracted through solvent extraction had been investigated comprehensively for their insecticidal activities. Phytochemicals are the chemicals derived from plants, which actually determine their biological activity. Alkaloids, phenols, saponins, carbohydrates, terpenoids, steroid, flavonoids, and volatile oils are the main classes of phytochemicals present in plants. Like other plants, *Lantana* spp. have also been comprehensively chemically profiled, especially for their essential oils. The literature shows that *Lantana* spp. solvent extracts are profiled or studied mainly for their pharmacological properties, such as their antioxidant, anti-microbial, and anti-tumour properties (Table 3) [30,31]. Although there is lack of studies reporting insecticidal and acaricidal activity of *Lantana* spp. extracts, a few studies have reported on the potential insecticidal and acaricidal activity of lantana solvent extracts [32,33,34,35,36]. *Lantana* spp. solvent extracts generally constitute polyphenols, including flavonoids and tannins [31]. In addition to these, saponins, alkaloids, terpenes, and sterols are also detected. There is a variation in the phytochemistry of solvent extracts from different parts of *Lantana* spp. [30,34]. Mansoori et al. [30] reported that flavonoids, phlobatannins, steroids, alkaloids, phenols, tannins, quinones, and coumarins, have been detected in both flower and leaf extracts of *L. camara*. However, they revealed that terpenoids, saponins, and anthocyanins were found only in flower extracts. Also, among all classes of phytochemicals, alkaloids, flavonoids, tannins, and coumarins are heavily present in leaf extracts, while phenols are heavily present in both leaf and flower extracts. The solvents used for extraction also determine the phytochemical composition of *Lantana* spp. extracts. Ayalew [33] revealed that steroids, flavonoids, tannins, glycerols, and saponins are present in all the three solvent extracts (methanol, ethanol, and ethyl acetate), but alkaloids are present in only methanol and ethanol extracts (Table 4). As already discussed, *Lantana* spp. solvent extracts constituting different compounds such as polyphenols, including flavonoids, tannins, sterols, saponins, alkaloids, glycosides, etc., unlike essential oils, are less explored for their insecticidal activities. For instance, methanol extracts of *L. camara* leaves have been reported to have significant fumigation and contact toxicity against three stored grain pests (*Sitosphilus oryzae*, *Callosbruchus chinensis*, and *Tribolium castaneum*). In another study, the methanol fraction of *L. camara* leaves has been reported to have significantly higher mortality and repellent activity against *Sitophilus zeamais*, followed by ethanol and ethyl acetate extracts [33]. Few studies have determined the insecticidal activity of solvent extracts of *L. camara* against mosquito species [37,38,39]. The acaricidal activities of *L. camara* have also been reported in the literature.For instance, 5% chloroform extracts of *L. camara* leaves have shown interesting termiticidal activity against the workers of *Microcerotermes beesoni* [15]. Ethanol extracts of this species are also reported to cause developmental defects (IGR activity) against the fourth instar larvae of *Helcoverpa armigera*, one of the polyphagous insects on field crops [36]. Figure 1 depicts the structure of different volatile and non-volatile constituents present in different fractions of *L. camara*.

Essential oils are secondary metabolites produced by different plant organs (flowers, buds, leaves, fruit, bark, seeds, wood, rhizomes, and roots) [43], which play a very significant role in plant defence and other signalling processes, including the attraction of pollinators and beneficial insects [44,45,46]. Genus *Lantana* is chemically more profiled for its essential oils. The essential oils of different species of *Lantana* throughout the world have been comprehensively characterised (Table 5). Various endogenous and exogenous factors, such as plant species, geographic location, climate, harvesting period, and extraction technique, influence the chemical makeup of the essential oils [47,48]. Studies show that β-caryophyllene, a sesquiterpene, is very consistent in the essential oils of *lantana* spp. [49,50,51,52,53] throughout the year, independent of sampling seasons, and acts as the main chemical marker for the *Lantana* genus [53]. However, there is a huge variation in β-caryophyllene in different species, in different geographical regions, and even in different parts of the same species [53,54]. Germacrene-D was the second most consistent essential oil component among *Lantana* spp. but is not present in a few species, such as *Lantana achranthifolia*, *Lantana aculeata*, *Lantana indica*, *Lantana involucrate*, *Lantana orangemene*, *Lantana salvifolia*, etc., and cannot be designated as a chemical marker for *Lantana* spp. β-cubebene, elixene, and β-phellandrene are solely detected in the essential oils of *Lantana canescens* and *Lantana radula* from Brazil, and can act as chemical markers for these species [53]. Considering *Lantana camara*, there is a huge variation of essential oil components in different parts of the plant (leaves, flowers, stems, and fruit). The essential oil from the leaves, flowers, and fruit of *L. camara* predominantly contains hydrocarbon sesquiterpenes, followed by other components (oxygenated sesquiterpenes, oxygenated monoterpenes, and hydrocarbon monoterpenes), while the essential oil of the *L. camara* stem contains oxygenated monoterpenes in higher amounts, followed by other components [54]. (E)-β-caryophyllene and α-humulene were the main components of leaf and flower essential oils. In addition, leaf essential oil also contains monoterpenes, such as α-pinene and sabinene, whereas flower essential oil contains sabinene and linalool (monoterpenes). Further, the composition of the fruit and stem essential oils of *L. camara* differ a bit from that of its leaves and flowers. The essential oils of fruit and stems predominantly contain sesquiterpenes, representing 41–92% and 23.9–81.6%, respectively [54]. Neral and geranial are present in higher amounts in the essential oil of fruit in August. Palmitic acid represents a major constituent of *L. camara* essential oil from South China and Northern India. The stem essential oil of *L. camara* is dominated by higher amounts of germacrene-d (31%) in South China and by palmitic acid (32.7%) in Northern India [55,56]. In contrast to this, Ivorian *L. camara* has been reported to represent a unique chemotype with high thymol content in comparison to the essential oils of *L. camara* growing in other countries [57]. Studies have shown that there is a huge seasonal variation in the essential oils of *L. camara* [57,58]. Zoubiri and Baaliouamer [57] reported that sesquiterpene hydrocarbons (β-caryophyllene and α-zingiberene) are found in higher amounts in March. Similarly, a huge seasonal fluctuation of thymol (an oxygenated monoterpene) concentration in the essential oils of different parts of *L. camara* has been reported [57]. This seasonal variation of some components indicated an environmental influence on the essential oil composition of *L. camara*. The above terpenoid compounds present in the essential oils, whether monoterpenes or sesquiterpenes, are synthesised mainly either via the methylerythritol 4-phosphate (MEP) pathway or mevalonate-dependent (MVA) pathway, and show potential insecticidal activity, especially against stored grain pests, due to their volatile nature [58]. The use of essential oils extracted from aromatic plants to manage insect pests have increased considerably due to their repellent, insecticidal, antifeedant, growth inhibitor, oviposition inhibitor, etc., properties on a variety of insect pests [59,60,61,62,63]. Aromatic plant families, viz., asterace, cupressaceae, lamiaceae, lauraceace, rutacea, myrtaceae, piperaceae, and poaceae, have been extensively exploited for their essential oils for insecticidal activities [61,64,65,66,67]. Due to the volatile nature of essential oils, these are most commonly investigated against stored grain insect pests, especially coleopteran insect pests [58]. Similarly, the insecticidal properties of the essential oils of *L. camara* against stored insect pests has been reported [57,68,69,70,71,72]. A study has shown that *L. camara* essential oil, with caryophyllene (69.96%) as its main compound, has significant insecticidal (fumigation, contact, and repellent) activity against *Tribolium castaneum*, *Lasioderma serricorne*, and *Callosobruchus chinenis.* A study has shown that there is a variation in the insecticidal activity and persistence of the essential oils of *Lantana camara* from different seasons against *Sitophilus granarius*, as there is a variation in the chemical composition of *Lantana camara* in different seasons [69]. The flower essential oil of *L. camara* is reported to have higher contact toxicity against *Sitophilus granaries* than the leaf essential oil of the same species [57]. This can be due to the higher thymol concentration in the flower essential oil of this species, as it is reported to have significant insecticidal activity [73,74]. Moreover, oxygenated monoterpenes, which are in high proportion in the flower essential oil of *L. camara*, could be one of the reasons for the higher insecticidal activity against *S. granaries*, as these molecules are frequently attributed to have insecticidal activities [61,75,76]. This concludes that the insecticidal activities of *L. camara* depend on the proportion of different components which vary in different seasons, regions, and parts of the plant. *L. camara* essential oil is also reported to have larvicidal and repellent activities against different mosquito species of medical and veterinary significance [25,77,78,79,80,81,82]. A liquid vaporizer based on the leaf essential oil of *L. camara* has been reported to have significant repellent activities against mosquitoes [78]. In addition to this, the essential oil of *L. camara* possesses insecticidal activities against other insect pests of medical, veterinary, and forestry significance. For instance, in addition to larval and pupal toxicity, the essential oil of *L. camara* has caused severe developmental defects in *Chrysomya megacephala*, a calliphorid insect pest [83]. Major forest defoliators, viz., *Hyblaea puera*, *Eligma narcissus*, and *Atteva fabriciella*, are highly susceptible to the essential oil of *L. camara* and a few of its major constituents, like caryophyllene II oxide and aromadenrene II oxide [84].

## 4. Mechanism of Action

Lantana is rich in diverse phytoconstituents like terpenoids, alkaloids, flavonoids, and phenolic compounds, all of which exhibit potent insecticidal properties [102]. These compounds disrupt cellular respiration, induce oxidative stress, cause mitochondrial damage, and trigger apoptosis at the molecular level, leading to insect death [103]. Additionally, Lantana’s antifeedant properties interfere with insect gustatory receptors, altering neurohormonal signalling and modulating neurotransmitter synthesis, drastically reducing feeding behaviour [104]. As a fumigant, Lantana’s essential oils target the nervous system by inhibiting acetylcholinesterase (AChE), blocking nerve impulse transmission and causing neurotoxicity, paralysis, and death [69]. With its multifaceted modes of action like oxidative stress, neurotoxicity, and feeding inhibition, Lantana offers promising potential as a natural insecticide for eco-friendly pest management (Table 6).

### 4.1. Insect Mortality

Lantana phytochemicals, including linalool and various alkaloids, disrupt insect nervous system function by interfering with key neurotransmitter pathways, leading to neurotoxic effects that result in paralysis and death [109]. Specifically, these compounds inhibit GABAergic and cholinergic signaling, causing severe disruptions in neural communication. The inhibition of GABA receptors results in the hyperexcitability of neurons, which manifests as uncontrolled muscle contractions and spasms, ultimately leading to paralysis [110]. Linalool, a prominent monoterpene in Lantana essential oils, specifically inhibits GABA receptor activity by binding to the receptor’s active site. This action prevents GABA, the primary inhibitory neurotransmitter in the insect central nervous system, from exerting its calming effects, leading to continuous neuronal firing and heightened excitability. Normally, GABA binding induces chloride ions (Cl^−^) to enter neurons, hyperpolarizing the membrane and preventing over-excitation. However, when GABAergic inhibition is lost, uncontrolled nerve firing ensues, resulting in tremors and uncoordinated movements. Prolonged neural hyperactivity particularly affects respiratory muscles, leading to paralysis and death [111]. In addition, alkaloids act as inhibitors of acetylcholinesterase (AChE), the enzyme responsible for breaking down acetylcholine (ACh) in the synaptic cleft. By binding to AChE’s active site, these alkaloids prevent the breakdown of acetylcholine, causing its accumulation and the overstimulation of acetylcholine receptors. This overstimulation results in the continuous activation of cholinergic neurons, leading to constant muscle contraction, twitching, and convulsions. Eventually, the excessive muscular stimulation culminates in paralysis and death, often due to respiratory failure or muscular exhaustion [112,113].

The oxidative stress induced by terpenoids and flavonoids in Lantana results in extensive mitochondrial damage, lipid peroxidation, and DNA oxidation, which ultimately disrupts cellular function and leads to insect death [114,115]. This oxidative imbalance occurs when reactive oxygen species (ROS) production exceeds the insect’s antioxidant defences, making oxidative stress one of the primary mechanisms by which Lantana phytochemicals cause insect mortality [116]. Key compounds, such as the pentacyclic triterpenoid lantadene A and flavonoids, are central to this process. Lantadene A, found in Lantana, induces oxidative stress by triggering an overproduction of ROS, including superoxide anions (O_2_^−^), hydrogen peroxide (H_2_O_2_), and hydroxyl radicals (•OH) [117]. These ROS primarily target the mitochondria, the energy-producing organelles of the cell. When ROS levels become excessive, they disrupt the electron transport chain (ETC), causing mitochondrial membrane depolarization and a significant reduction in ATP synthesis, which is essential for energy production [118]. The collapse of mitochondrial membrane potential leads to the release of pro-apoptotic factors such as cytochrome c, initiating programmed cell death (apoptosis). Moreover, the ROS generated by lantadene A induce lipid peroxidation, damaging the insect’s cellular membranes by destroying phospholipids, which increases membrane permeability and disrupts critical cellular functions. Additionally, ROS can oxidize proteins and DNA, further impairing cell function and triggering apoptosis. DNA oxidation, in particular, disrupts the cell cycle and promotes programmed cell death [119,120]. Flavonoids in Lantana also play a role in generating ROS. These compounds can reduce molecular oxygen to produce superoxide radicals, similar to lantadene A’s mechanism. By targeting mitochondria, flavonoids impair oxidative phosphorylation, leading to an energy deficit in insects. Furthermore, flavonoids can inhibit key antioxidant enzymes, such as superoxide dismutase (SOD), catalase (CAT), and glutathione peroxidase (GPx), which normally work to neutralize ROS [30,121]. The inhibition of these enzymes exacerbates ROS accumulation, causing further cellular damage and contributing to insect mortality.

Insect mortality is also induced by phytochemicals, resulting in the disruption of cellular structure and triggering apoptosis. This process involves damage to cell membranes, ionic imbalance, and the activation of apoptosis-related enzymes. Terpenoids, being lipophilic, integrate into insect cell membranes and disrupt their fluidity and permeability. By interacting with the lipid bilayer, terpenoids compromise membrane integrity, leading to ion leakage and osmotic imbalance, causing a loss of essential ions, such as Ca^2+^, and nutrients [122,123]. Essential oils like linalool further enhance membrane permeability by forming pores, amplifying the disruption of the membrane’s selective barrier function [124]. This membrane damage triggers a rapid influx of calcium ions into the cytoplasm, which activates a cascade of enzymes, including calpains and caspases, responsible for degrading cellular components. The resulting calcium overload also impairs mitochondrial function and ATP synthesis, depleting the energy required for cellular survival. In addition, calcium activates phospholipases that degrade membrane lipids, exacerbating cellular damage [125]. The loss of mitochondrial membrane potential and the release of cytochrome c into the cytoplasm initiate the intrinsic (mitochondrial) pathway of apoptosis. Cytochrome c binds to apoptotic protease activating factor-1 (Apaf-1), forming the apoptosome, which activates caspase-9 and, subsequently, downstream effector caspases such as caspase-3 and caspase-7. These caspases cleave structural proteins, degrade DNA, and dismantle cellular organelles [126]. The apoptosis pathway ensures the systematic breakdown of cellular components, eliminating damaged or dysfunctional cells. In insects, this results in the destruction of vital tissues and organs, leading to death. The combined effects of membrane disruption, ionic imbalance, and the activation of apoptotic pathways efficiently target multiple organ systems, ensuring insect death [127]. This mechanism of inducing apoptosis is a critical feature of Lantana’s insecticidal action, delivering lethal effects even at lower concentrations of phytochemicals [117].

### 4.2. Antifeedant Mechanism

Lantana phytochemicals exert antifeedant activity by targeting key biochemical pathways in insects, disrupting their ability to feed, digest, and absorb nutrients, ultimately leading to physiological and developmental damage. Insects depend on intricate mechanisms for feeding, involving the detection and processing of chemical cues from their food sources [128]. A primary mechanism behind this antifeedant activity involves the interaction of compounds with insect gustatory receptors (GRs), mainly located on the mouthparts. These GRs play a critical role in the insect’s ability to evaluate food by detecting various chemical signals, including bitter or deterrent compounds [129]. When volatile terpenoids like linalool make contact with these receptors, they bind specifically to the GRs responsible for detecting unpleasant or toxic chemicals, leading to an aversion response. This interaction triggers the insect to reject the plant as a food source. Upon binding to the gustatory receptors, the signaling pathways associated with feeding behaviour are disrupted. This causes the insect to perceive the plant as unpalatable or harmful, leading to a significant reduction in feeding, and in some cases, feeding may be entirely stopped after only a few bites [130,131]. This response is highly effective in disrupting insect–host relationships, as insects quickly learn to associate Lantana with an unpleasant feeding experience. The underlying mechanism involves changes in the firing rates of neurons linked to these gustatory receptors. For instance, linalool interacts with ion channels on gustatory receptor cells, altering normal neuronal activity. This disruption in neuronal firing sends negative signals to the insect’s central nervous system (CNS), prompting it to cease feeding [132]. As the insect’s sensory perception of the plant is disturbed, it is less likely to continue feeding on the plant, leading to reduced plant damage and making Lantana extract a powerful deterrent against herbivorous insects.

A crucial aspect of Lantana’s antifeedant activity lies in its inhibition of essential digestive enzymes within the insect gut. Phytochemicals like flavonoids and tannins are particularly effective at disrupting these enzymes, impairing the digestion and absorption of nutrients, which leads to malnutrition, weakened physiological functions, and eventually, death [133]. One such enzyme is amylase, responsible for breaking down carbohydrates into simpler sugars. Flavonoids, including quercetin and kaempferol found in Lantana, inhibit amylase activity by binding to its active sites, preventing the enzyme from catalysing the conversion of polysaccharides (such as starch) into monosaccharides [134], which serve as vital energy sources for insects. This inefficiency in carbohydrate digestion leads to an energy deficit, weakening the insect’s metabolic processes. In addition to amylase, proteases, which break down proteins into amino acids essential for insect growth and reproduction, are also targeted by Lantana phytochemicals. Protease inhibitors in Lantana block these enzymes, making it difficult for insects to digest proteins effectively. This disruption in protein digestion hampers tissue development and reproductive functions, contributing to stunted growth and reduced fecundity [135,136]. Furthermore, lipases, enzymes responsible for breaking down fats into fatty acids, are inhibited by Lantana compounds. By reducing lipase activity, insects are deprived of essential lipids required for energy storage and the structure of cell membranes. This reduction in lipid availability exacerbates the effects of nutrient starvation, further impairing growth and reproductive capacity [137]. Tannins, another group of Lantana phytochemicals, bind to proteins in the insect gut, including both digestive enzymes and dietary proteins [94]. This interaction forms insoluble complexes that cannot be broken down or absorbed by the insect, leading to reduced protein availability [138]. The resultant nutrient deprivation severely impairs the insect’s ability to grow, reproduce, and survive, making Lantana a highly effective deterrent against herbivorous insects.

### 4.3. Fumigant Action

Lantana’s volatile compounds also demonstrate potent neurotoxic effects, primarily through the inhibition of acetylcholinesterase (AChE), an enzyme vital for regulating nerve impulses. Compounds like camphor and linalool specifically target the nervous system by inhibiting AChE, leading to the accumulation of acetylcholine (ACh) at the synapses, which causes continuous nerve stimulation, ultimately resulting in paralysis and rapid death [17,57,139]. Under normal conditions, AChE breaks down acetylcholine after it has transmitted a signal between nerve cells. However, when compounds like camphor and linalool inhibit AChE, acetylcholine remains active, continuously activating its receptors and causing sustained muscular contraction and uncontrolled nerve signaling. This overstimulation of nerve impulses leads to paralysis, convulsions, and eventual death, as the respiratory muscles fail due to the lack of regulation [140]. Lantana’s volatile compounds achieve this effect by binding to the active site of AChE, preventing it from hydrolysing acetylcholine into choline and acetate. As a result, acetylcholine builds up at neuromuscular junctions, extending its interaction with acetylcholine receptors (AChRs) and triggering continuous nerve signals. This persistent signaling exhausts the nervous system, causing neural exhaustion and paralysis [141].

One of the key factors enhancing the insecticidal effectiveness of Lantana’s compounds is their volatility. Essential oils like linalool, camphor, and eucalyptol are highly volatile, meaning they easily vaporize and disperse in the air. This property makes them highly effective fumigants, especially in enclosed environments where the concentration of these compounds can build up to lethal levels [142]. The volatility of essential oils allows them to penetrate the insect’s protective barriers, such as the cuticle, and reach critical sites like the spiracles and nervous system [143]. In environments like grain storage facilities, these volatile compounds accumulate, ensuring prolonged exposure to the target insects, increasing the likelihood of lethal outcomes. Once these volatile compounds, such as linalool and camphor, enter the insect’s system, they rapidly diffuse across membranes and tissues, interacting with lipid structures. This enables them to disrupt gas exchange through the spiracles and interfere with nerve function [144]. In confined spaces, the higher concentration of these compounds amplifies their effects, leading to more pronounced inhibition of key enzymes like acetylcholinesterase (AChE) and cytochrome c oxidase [145]. As a result, the buildup of acetylcholine at neuromuscular junctions and the disruption of respiratory enzymes results in faster mortality rates in insects exposed to Lantana essential oils in such environments.

While linalool and camphor are the primary volatile compounds responsible for Lantana’s fumigant action, other phytochemicals also contribute to enhancing its insecticidal activity. Lantadene A, a triterpenoid, induces oxidative stress in insect cells by generating reactive oxygen species (ROS). The excessive ROS production disrupts the cellular redox balance, causing oxidative damage to proteins, lipids, and nucleic acids. This oxidative stress impairs cellular metabolism, accelerating the insect’s death, particularly when combined with respiratory or neural disruption caused by other compounds [117]. Another important phytochemical is eucalyptol (1,8-cineole), which exhibits neurotoxic properties similar to those of linalool and camphor. Eucalyptol further enhances the overall fumigant effect by intensifying the respiratory and neural disruption initiated by other volatile compounds [146]. The combined action of these phytochemicals amplifies Lantana’s insecticidal efficacy, making it a powerful natural alternative for pest control.

## 5. Molecular Docking Studies

Molecular docking has emerged as a crucial computational tool to elucidate the mechanisms by which plant-derived compounds exert insecticidal activity. The rich phytochemical profile of *L. camara* has long been recognized for its potential in pest control, owing to the presence of a variety of bioactive compounds such as terpenoids, flavonoids, and essential oils. These compounds exhibit multiple modes of action, including neurotoxic effects by inhibiting acetylcholinesterase (AChE), interference with digestive enzymes, and disruption of insect receptors. One of the most studied insecticidal mechanisms of Lantana compounds is their ability to inhibit acetylcholinesterase (AChE), an enzyme crucial for regulating neural transmission by hydrolysing acetylcholine at synapses. Lantana compounds, especially β-caryophyllene and linalool, are widely recognized for their insecticidal potential, primarily due to their inhibition of acetylcholinesterase (AChE), a key enzyme in neural function. β-caryophyllene, derived from *Lantana indica*, has demonstrated a stable interaction with AChE, as reflected by its docking score of −67.7, which suggests significant neurotoxic properties [147]. Similarly, linalool from *Lantana* essential oils shows stable binding with AChE, effectively blocking acetylcholine degradation and resulting in sustained nerve excitation [148]. Lantadene A, a principal compound in *L. camara*, interacts strongly with AChE from *O. taurus* and *L. decemlineata*, with low binding energies of −87.4555 kcal/mol and −98.6 kcal/mol, respectively [149]. Molecular docking has identified nine interaction models between these ligands and their macromolecular targets. Trans-caryophyllene, in particular, showed a binding affinity of −8.3 kcal/mol with AChE, while linalool exhibited a slightly higher energy requirement at −5.5 kcal/mol, suggesting that trans-caryophyllene may bind more spontaneously and stably to AChE [150]. Both compounds share a similar binding position, indicating their comparable potential as AChE inhibitors. Moreover, β-caryophyllene also exhibited a strong interaction with *A. gambiae*, with a binding affinity of −9.5 kcal/mol [151], reinforcing its insecticidal efficacy. Further studies highlighted e-caryophyllene’s binding affinity of −9.3 kcal/mol against AChE [152], while other compounds such as camphene, humulene, and γ-cadinene displayed binding affinities of −7.2, −9.2, and −9.7 kcal/mol, respectively, against mosquito juvenile hormone-binding protein [153]. This broad spectrum of interactions underscores the versatility of Lantana phytochemicals as potential bioinsecticides, targeting various proteins critical to insect survival.

Lantana compounds not only disrupt neural pathways but also inhibit digestive enzymes like amylases, proteases, and lipases, essential for nutrient absorption in insects. Docking studies revealed that compounds such as pectolinaringenin, naphthalene, and gamolenic acid exhibit strong interactions with digestive enzymes, indicating their larvicidal potential [154,155,156,157]. Kaempferol, a flavonoid in Lantana, also showed strong binding to these enzymes, impairing digestion by preventing substrate binding [158]. This dual-action mechanism highlights Lantana’s effectiveness in disrupting both neural and digestive functions in insects. Lantana phytochemicals also target critical proteins involved in cellular metabolism and signaling, such as Peptide Deformylase (PDF), which removes the N-formyl group from N-terminal methionine during translation. Docking studies revealed that compounds like eicosapentaenoic acid and loliolide from Lantana bind strongly to PDF, disrupting protein synthesis and leading to insect mortality [30]. These compounds also interact with Mitogen-Activated Protein Kinase (MAPK1) and sucrose hydrolase (SUH), further impairing cell signaling and carbohydrate metabolism, contributing to cell death.

The structure–activity relationship (SAR) analysis of Lantana phytochemicals provides valuable insights into how their chemical structures influence their binding affinities and insecticidal properties. SAR studies help identify the functional groups and molecular motifs responsible for interacting with target proteins, thus allowing for a more focused approach to bioinsecticide development. For instance, the high docking score of β-caryophyllene with AChE (−67.7) can be attributed to its hydrophobic interactions and stable conformation [147]. The bicyclic structure of β-caryophyllene allows it to fit snugly into the enzyme’s active site, forming non-covalent interactions that prevent the binding of acetylcholine. This structural feature is crucial for its AChE inhibitory potential, making it a promising neurotoxic agent. Similarly, kaempferol’s flavonoid backbone with hydroxyl groups facilitates hydrogen bonding with the active sites of digestive enzymes, thereby blocking their activity [149]. This structural feature explains kaempferol’s potent insecticidal activity, as it effectively inhibits nutrient absorption in insects. Linalool and camphor, both monoterpenoids found in Lantana essential oils, also exhibit strong binding affinities due to their structural properties. Their small, hydrophobic nature allows them to easily penetrate cell membranes and access enzyme active sites, where they disrupt enzymatic function. The structural similarity of linalool and camphor with acetylcholine enables them to competitively inhibit AChE, leading to neurotoxic effects.

Molecular docking provides predictions of binding affinities, but ADMET (Absorption, Distribution, Metabolism, Excretion, and Toxicity) studies are essential to evaluate the pharmacokinetics and safety of Lantana phytochemicals as insecticides. Kaempferol, stigmasterol, and lantadene have shown potential as bioinsecticides based on enzyme interactions [149], but toxicity can vary with application methods and environmental factors. For example, kaempferol might have lower toxicity when used topically, while lipid-soluble compounds like stigmasterol and lantadene could accumulate in insect tissues, leading to higher toxicity. ADMET studies confirm their favourable bioavailability, though their effects on non-target species and bioaccumulation risks require further investigation.

## 6. Conclusions

*L. camara* is a medicinal plant that has gained recognition for the presence of diverse phytochemicals, primarily and secondarily metabolites, alkaloids, flavonoids, tannins, saponins, and terpenoids. These compounds contribute fully to its therapeutic potential, hence making *L. camara* a very interesting subject in pharmacology studies. There is substantial research that has been exhibited on various parts of the plant, mainly consisting of leaves, stems, and fruit with rich biochemicals. For instance, flavonoids, tannins, and saponins are highly concentrated in the extracts of leaves; notable amounts of oleanolic acid were reported in roots. The composition of essential oils from *L. camara* is highly heterogeneous and variable according to the plant part, geographical area, or the species considered. Hydrocarbons and oxygenated terpenes account for the major constituents of the essential oil derived from the leaves and flowers of the plant, with β-caryophyllene being present in a large percentage. Among the main compounds identified, β-caryophyllene remains one of the most active of the common markers identified in the Lantana genus apart from lantadene A, B, C, and D. This phytoconstituent contributes to the insecticidal potency of the plant. Scientists have studied this chemical and agreed that it had high fumigant and contact toxicities against many insects, such as *Tribolium castaneum* and *Lasioderma serricorne*.

On the other hand, solvent extracts, especially the methanol extracts of the leaves, have shown significant fumigant and contact toxicity against the pests of stored grains, though the usage potential of such extracts remains less exploited compared to essential oils. Molecular docking studies have explained the mechanism of action of *L. camara*’s phytochemicals being insecticidal. One of the more important mechanisms is the inhibition of an enzyme known as acetylcholinesterase (AChE), critical for the transmission of neural impulses. Such compounds as β-caryophyllene and linalool could show strong binding affinities toward AChE, preventing acetylcholine degradation, thus leading to prolonged nerve excitations. Such a neurotoxic function would explain the mechanism whereby *L. camara* exerts insecticidal activity. 

Further investigations of the binding affinities of other terpenoids including trans-caryophyllene and E-caryophyllene reveal that they are potent AChE inhibitors, thus shedding more light on the potential of the plant species as a naturally occurring insecticide. The various phytochemicals responsible for the different active compounds capable of binding to the various physiological systems of insects justify the science behind the compound’s functioning through various modes to produce high insecticidal activity. With a rich phytochemical profile coupled with significant biological activity, there is potential for the use of *L. camara* as an insecticidal agent. Linalool and β-caryophyllene are two promising natural compounds present in various botanicals, including *Lantana camara*, that can be used as a source of sustainable alternate insecticides. Being a monoterpene alcohol, linalool has been shown to be neurotoxic, which inhibits AChE in the insects, thereby causing paralysis due to disrupted nerve transmission. It is also repellent and is active against a broad spectrum of pests. The sesquiterpene β-Caryophyllene acts on the endocannabinoid system of the insect; it binds to CB2 receptors and intervenes in normal physiological functions, leading to neurotoxic effects along with repellency. Both are biodegradable in nature, thus less toxic to humans and animals. They have lower environmental hazards as compared to chemical synthetic products, and there also is low resistance development. This makes them potent options when put together, as combined, they can exhibit synergies, thus enhanced pest control effectiveness and lower chances of insect resistance buildup. These botanicals fulfil the increasing demand of sustainable, eco-friendly pest management in agriculture. Further studies and optimization are needed, but linalool and β-caryophyllene-containing botanicals have very good potential as a safe and effective insecticide, contributing towards naturalistic pest management and the reduction of chemical pest inputs that threaten health and the environment. Its highlight lies in the production of sustainable pest management strategies, especially synthetic insecticides substitutes. As more research continues to advance to the farthest reaches of its bioactive compounds and specific modes of action, *L. camara* will have immense utility within agricultural pest control and integrated pest management systems. However, from now on, to improve the extraction and utilization of the insecticidal activity of this compound, eco-friendly solutions for pest-related challenges may be provided.

## Figures and Tables

**Table 1:** Taxonomic classification of *Lantana camara* [4].

Kingdom	Plantae
Subkingdom	Tracheobionta
Super division	Spermatophyta
Division	Magnoliophyta
Class	Magnoliopsida
Order	Lamiales
Family	Verbenaceae
Genus	Lantana
Species	*Lantana camara* Linn

**Table 2:** Geographical distribution, habitat adaptation, and morphological characterization of *L. camara*.

Features	Explanation
Native	Tropics of South and Central America
Synonym	*Lantana scabrida*, *Camara vulgaris*
Plant category	Annuals or biennials, perennials, shrubs, ground covers
Distribution	35° N and 35° S latitudes
Conservation status	Alien
Foliage characteristics	Aromatic, evergreen, noxious
Foliage colour	Dusky green
Flower	Long permanent, attractive, uncommon with yellow, orange, and pink colours
Tolerances against environmental stresses	Pollution, wind, drought, slope, heat, and humidity
Propagation method	Stem cuttings
Pollinators	Thrips and Lepidopteran species
Light requirement	Sun to full sun
Plant characteristics	Toxic
pH requirement	4.5–8.5
Temperature requirement	Intolerant to freezing
Annual rainfall requirement	1000–4000 mm
Soil	Sandy to clay loam
Water	Semi-arid to normal
Light conditions	Prefers unshaded habitats; can tolerate some shade
Altitude	Less than 2000 m above sea level

**Table 3:** Table highlighting potent major therapeutic activities exhibited by *L. camara*.

S. No.	Activity Reported	Plant Part Assessed	Fractions Screened	References Cited
1.	Antioxidant	Leaves	Methanol	[19]
2.	Antifungal	Leaves	Methanol	[20]
3.	Anti-microbial	Flowers, leaves, roots, stems	Chloroform	[19,20]
4.	Wound healing	Leaves	Ethanol	[19,20]
5.	Anti-viral	Leaves	Methanol	[20]
6.	Anti-parasitic	Leaves	Methanol	[21]
7.	Antipyretic	Leaves	Ethanol, ethyl acetate	[22]
8.	Anti-ulcerogenic	Leaves	Methanol	[20,21]
9.	Antibacterial	Leaves	Methanol	[20]
10.	Anti-inflammatory	Leaves	Aqueous	[22]
11.	Anti-hyperglycaemic	Leaves	Methanol	[21,22]
12.	Anti-motility	Leaves	Methanol	[22]
13.	Mosquito control	Leaves	Essential oil	[23]
14.	Anti-mitotic	Flowers	Petroleum ether, chloroform, ethanol, aqueous	[19,21]
15.	Anti-filarial	Leaves	Methanol	[19]
16.	Anti-mutagenic	Leaves	Methanol	[24]
17.	Anti-cancer and Anti-proliferative	Leaves	Methanol	[20,24]
18.	Anti-helmintic	Leaves, stems, roots	Methanol	[21,24]

**Table 4:** Interspecies variation in the major phytoconstituents present in different solvent fractions of *Lantana* spp.

S. No.	Lantana Species	Plant Parts Assessed	Fractions Screened	Categories of Phytoconstituents Reported	Ref.
1.	*L. camara* L.	Leaf	Methanol	Alkaloids, phenols, flavonoids, tannins, coumarins, steroids, cardiac glycosides	[30]
		Flower	Methanol	Phenols, flavonoids, anthocyanin, tannins, alkaloids	
2.	*L. camara* L.	Leaf	Methanol	Steroids, flavonoids, glycosides, tannins, saponins	[33]
			Ethanol	Steroids, alkaloids, flavonoids, saponins	
			Ethyl acetate	Fixed oils, glycosides, flavonoids	
3.	*L. camara* L.	Leaf	Petroleum ether		[34]
			Methanol	Triterpenoids, proteins	
			Water	Resins, carbohydrates, proteins	
			Methanol/water (100:10)	Triterpenoids, steroids, resins	
			Hexane	Flavonoids, tannins, fixed oils	
			chloroform	Triterpenoids, steroids, lactones	
			n-butanol	Flavonoids	
4.	*L. camara* L.	Leaf	Methanol	Tannins, saponins, terpenoids, glycosides, alkaloids	[31,34]
		Stem	Methanol	Saponins, flavonoids, terpenoids, alkaloids	
		Fruit	Methanol	Tannins, saponins, flavonoids, terpenoids	
5.	*L. camara* L.	Whole plant	Methanol	Tannins and saponins	[40]
6.	*Lantana rhodesiensis*	Leaf	Methanol	Polyphenol, flavonoids, terpenes, tannins	[31]
		Stem	Methanol	Polyphenols, tannins	
		Root	Methanol	Terpenes, saponins	
7.	*Lantana montevidensis*	Leaf	Ethanol	Polyphenolic compounds (phenolic acids and flavonoids)	[41]
			Aqueous	Polyphenolic compounds (phenolic acids and flavonoids)	
8.	*Lantana montevidensis*	Leaf	Ethanol	Phenol (32.5%) and flavonoids (7.1%)	[42]
		Root	Ethanol	Phenol (18.2%) and flavonoids (3.2%)	

**Table 5:** Interspecies variation in the major phytoconstituents present in essential oils of *Lantana* spp.

S. No.	Lantana Species	Country	Plant Part Assessed	Major Volatiles Reported	Ref.
1.	*L. camara*	India	-	α-pinene (15.3%), caryophyllene (15.28%), eucalyptol (7.8%), camphene (6.05%), caryophyllene oxide (5.33%), β-pinene (4.8%), geranyl acetate (4.3%)	[83]
2.	*L. camara*	Algeria	Aerial parts	β-Caryophyllene (26.3–46.7%), α-Acoradiene (3.7–15.3%), caryophyllene oxide (2.2–18.8%), β-Elemene (1.3–7.2), germacrene-D (0.8–4.3%), α-Humulene (0.4–4.7%)	[69]
3.	*L. camara*	Bregbo, Ivory Coast	Leaf	(E)-β-caryophyllene (24.4–39.9%), α-humulene (10.1–20.5%), α-pinene (1.1–6.5), sabinene (2.5–10.9%), thymol (0.00–18.4%), γ-muurolene (2.3–7.4%)	[54]
			Flower	(E)-β-caryophyllene (19.2–36.6%), α-humulene (8.5–19.9%), thymol (0.00–34.3%), sabinene (0.2–5.8%), γ-muurolene (2.0–7.7%), linalool (0.8–3.1%)	
			Stem	E-β-caryophyllene (7.3–25.3%), α-humulene (3.2–15.4%), thymol (0.00–41.4%), linalool (0.2–9.2%), E-β-farnesene (1.4–6.9%)	
			Fruit	E-β-caryophyllene (11.2–36.9%), thymol (0.00–27.6%), α-humulene (5.6–18.3%), linalool (0.7–6.0), E-β-farnesene (1.8–6.3)	
4.	*L. camara*	North India (Dehradun)	Leaf	(E)-β-caryophyllene (23.3%), α-humulene 11.5%, germacrene-D (10.9%), davanone (7.3%)	[85]
5.	*L. camara*	Brazil	Leaf	Germacrene-D (19.8%), *E*-caryophyllene (19.7%), bicyclogermacrene (11.7%), α-humulene (9.3%)	[52]
6.	*L. camara*	South China	Leaf	Germacrene-D (20%), trans-caryophyllene (14.8%)	[56]
7.	*L. camara*	Northeast India	Leaf	cis-davanone (47.8%), (E)-β-caryophyllene (10.3%)	[86]
8.	*L. camara*	Bangladesh	Leaf	(E)-β-caryophyllene (13.57%), α-caryophyllene (11.76%), germacrene-D (10.88%), isocaryophyllene (9.59%), γ-muurolene (6.85%)	[87]
9.	*L. camara*	Benin	Leaf	Sabinene (38.81%), 1,8-cineole (28.90%)	[88]
10.	*L. camara*	Kerala, India	Leaf	Caryophyllene (69.96%), isoledene (12.00%), α-Copaene (4.11%)	[68]
11.	*L camara*	Dehradun, India	Leaf	Trans-caryophyllene (13.95%), sabinene (8.28%), (E)-citral (6.9%), bicyclogermacrene (9.77%), α-curcumene (8.57%)	[89]
12.	*L. camara*	Venezuela	Leaf	Germacrene-D (31%), (E)-β-caryophyllene (14.8%)	[90]
13.	*Lantana canescens*	Brazil	Leaf	β-Caryophyllene (43.9%), β-Cubebene (10.1%), Elixene (8.6%), β-Phellandrene (6.1%)	[53]
14.	*Lantana radula*	Brazil	Leaf	β-Cubebene (31.0%), β-Caryophyllene (20.8%), Elixene (10.0%), β-Phellandrene (6.1%)	
15.	*L. radula*	Brazil	Leaf	*E*-Caryophyllene (25.3 ± 5.47%), germacrene-D (17.6 ± 1.21%), *E*-nerolidol (19.0 ± 3.56%), phytol (29.2 ± 5.23%)	[52]
16.	*L. achranthifolia*	Mexico	Aerial parts	Carvacrol (30.6%), alpha-bisabolol (11.2%), isocaryophyllene (10.7%)	[91]
17.	*L. aculeata*	India	Leaf	Caryophyllene (4.62%), α-phellandrene (18.89%)	[18]
18.	*L. hirta*	Costa Rica	Leaf	1-octen-3-ol (64.6%), germacrene-D (24.5%), (*E*)-caryophyllene (10.9%)	[92]
19.	*L. indica*	North India	Leaf	β-caryophyllene (8.4%), spathulenol (12.8%),	[93]
20.	*L. indica*	India	Leaf	Sabinene (14.8%), δ-3-carene (15.6%), α-humulene (17.8%), (E)-nerolidol (9.2%)	[94]
21.	*L. involucrata*	Cuba	Leaf	Alpha-eudesmol (24.9%), β-caryophyllene (10.6%)	[95]
22.	*L. fucata*	Brazil	Leaf	Caryophyllene oxide (27.9%), gossonorol (18.2%), β-caryophyllene (12.3%), bulnesol (10.8%),	[96]
		Brazil	Leaf	β-elemene (27.1%), germacrene-D (11.8%), β-caryophyllene (7.7%)	[97]
23.	*L. orangemene*	India	Leaf	β-caryophyllene (2.61%), β-phellandrene (21.92%), geraniol (18.65%)	[98]
24.	*L. salvifolia*	Congo	Leaf	β-caryophyllene (11–18%), geranial (26–34%), neral (15–20%)	[99]
25.	*L. trifolia*	Rwanda	Leaf, flower, whole plant	Caryophyllene (11.3–14.3), germacrene-D (16.9–33%)	[100]
26.	*L. velutina*	Costa Rica	Leaf	*(E*)-caryophyllene (23.4%), limonene (21.4%), bicyclogermacrene (8.2%)	[92]
27.	*L. xenica*	Argentina	Aerial parts	β-caryophyllene (35.2%), cadinene (13.3%)	[101]

**Table 6:** Mode of action of different fractions of *L. camara* for insecticidal activity.

S. No.	Plant Fractions Assessed	Mode of Action	Pests Targeted	Ref.
	Solvent extracts (methanol)	Contact, fumigation, grain-protectant activity	*Sitophilus oryzae*, *Callsosbruchus chinensis*, and *Tribolium casteneum*	[17]
	Solvent extracts (petroleum ether, methanol, water, methanol/water (90:10), hexane, chloroform)	Termiticidal activity against worker termites	*Microcerotermes beesoni*	[24]
	Solvent extracts (ethanol)	Developmental effects	*Helicoverpa armigera* larvae	[36]
	Solvent extracts (methanol, ethanol, and ethyl acetate)	Repellent and adulticidal activity	*Sitophilus zeamais*	[33]
	Solvent extracts (chloroform, ethyl acetate, and methanol extracts)	Acaricidal activity	*Rhipicephalus* (*Boophilus*) *microplus*	[105]
	Solvent extracts (ethanol)	Larvicidal activity	*Anopheles arabiensis* and *Culex quinquefasciatus*	[39]
	Solvent extracts (aqueous and ethanol)	Larvicidal activity	*Aedes aegypti*	[38]
	Essential oil	Larvicidal activity	*Hyblaea puera Eligma narcissus* and *Atteva fabriciella*	[84]
	Essential oil	Contact, fumigation, and repellent activity	*Tribolium castaneum*, *Lasioderma serricorne*, and *Callosobruchus chinensis*	[68]
	Essential oil	Contact and repellent activity	*Sitophilus granarius*	[30]
	Essential oil	Insecticidal activity	*Sitophilus oryzae*, *Tribolium castaneum Tribolium castaneum*, *Rhyzopertha dominica*, and mosquito larvae of *Culex pipiens*	[106]
	Essential oil	Fumigation activity	*Sitophilus granarius*	[69]
	Essential oil	Fumigation activity	*Sitosphilus oryzae*	[35]
	Essential oil	Grain-protectant activity	*Sitophilus zeamais*	[72]
	Essential oil	Developmental effects	*Chrysomya megacephala*	[83]
	Essential oil	Larvicidal activity	*Culex pipiens*	[57]
	Essential oil, solvent extracts	Repellent activity	*Aedes albopictus*	[23]
	Essential oil	Adulticidal activity	*Aedes aegypti*, *Culix quinquefasciatus*, *Anopheles culicifacies*, and *Anopheles fluviatilis fluvialitis*	[37]
	Essential oil	Larvicidal and pupicidal activity	*Anopheles culicifacies*	[82]
	Essential oil	Larvicidal and repellent activity	*Anopheles subpictus*, *Aedes aegypti*, and *Culex quinquefasciatus*	[29]
	Essential oil	Larvicidal activity	*Aedes aegypti* (L.), *Aedes albopictus*, and *Culex quinquefasciatus*	[107]
	Essential oil, leaf extract	Repellent activity	*Aedes aegypti*	[108]

## Data Availability

Not applicable.
